# Journal response types and times: the outcomes of manuscripts finalised for submission by the University of the Free State School of Medicine medical editor, South Africa

**DOI:** 10.11604/pamj.2020.36.212.24175

**Published:** 2020-07-24

**Authors:** Gina Joubert, Theanette Mulder, Wilhelm Johannes Steinberg, Johan Botes

**Affiliations:** 1 Department of Biostatistics, Faculty of Health Sciences, University of the Free State, PO Box 339, Bloemfontein, 9300, South Africa,; 2 School of Medicine, Faculty of Health Sciences, University of the Free State, PO Box 339, Bloemfontein, 9300, South Africa,; 3 Department of Family Medicine, Faculty of Health Sciences, University of the Free State, PO Box 339, Bloemfontein, 9330, South Africa

**Keywords:** Peer-reviewed journals, submission, outcomes, journal response, expectations

## Abstract

**Introduction:**

health professionals are involved in research as researchers themselves and as supervisors to undergraduate and postgraduate students. Authors may have unrealistic expectations regarding journal submission and review processes. The study aimed to describe journal response types and times for manuscripts finalised for submission by the University of the Free State School of Medicine medical editor.

**Methods:**

this descriptive cohort study with an analytical component included all manuscripts finalised for submission to accredited journals by the medical editor, 2014-2017. Excel spreadsheets capturing all stages of the manuscript process were used to confidentially note information regarding submission and subsequent journal responses.

**Results:**

ninety-five manuscripts were submitted to 72 peer-reviewed accredited journals. The total number of submissions was 163. Only 46 (48.4%) manuscripts were accepted by the first journals submitted to. Rejected submissions (n=82) had a median journal response time of 15.5 days (range 0-381 days), with a third being sent for review. Nine manuscripts were accepted with no revisions needed. Accepted submissions (n=72) had a median of one round of revision (range 0-4 rounds), and a median time of 119.5 days (range 0-674 days) from submission to final acceptance.

**Conclusion:**

within our setting, half of first submissions were unsuccessful, but rejection usually occurred rapidly. Acceptance for publication occurred at a median time of 4 months after one round of revision. If health professionals were made aware of expected outcomes and response times, it may prevent authors from falling victim to the publication practices of predatory journals.

## Introduction

Health professionals are involved in research as researchers themselves and as supervisors to undergraduate and postgraduate students. For example, a research component in medical education has proven to be vital for the development of successful medical professionals [1,2]. Critical reading, analytical thinking, communication skills, self-directedness and a broadened perspective on the medical field are just some of the skills acquired [1-3]. With the current emphasis on evidence-based medicine, it is important to develop an investigative mindset early on in students´ tertiary education [3,4]. Many medical schools have developed either formal or informal programs to give students the opportunity to acquire research skills [3,4]. In South Africa, all postgraduate MMed students studying to register for specialisation fields are required to complete research projects [2]. Furthermore, universities have opted to give all postgraduate students the option of publication-ready dissertations, which significantly shortened completion time, and increased publication outputs [2]. Research publishing is imperative for scientific advancement, to develop students´ continued interest in research, for the supervisors´ status as an academic and/or scientific physician, and for subsidy income for academic institutions [5-8].

The cornerstone of research publishing is thorough peer review. As the editor in chief of the Journal of the American Medical Association stated in 2017 [9]: *sacrificing adequate and thoughtful peer review and editorial assessment is a mistake for research in medicine. Timely assessment and dissemination of medical research findings is certainly important, but for most articles, rushing to publication in days or weeks will not improve health outcomes*. Inexperienced authors such as postgraduate students or new health professionals, in particular, tend to have unrealistic expectations regarding the review process. These authors are inclined to withdraw submissions when there is no rapid journal response, or the journal requires modifications to the manuscript [6,10]. A danger exists that such authors may easily fall prey to predatory journals which offer tantalisingly short turnaround times ('*if we can have your manuscript in the next 7 days, it will appear in the next issue of our journal*' - a typical e-mailed solicitation) or seem to be willing to publish manuscripts on any topic. Beall [11] was of the opinion that young researchers who are 'unfamiliar with the scholarly communication ecosystem' are specifically targeted by predatory journals or publishers. As reviewers for journals, who often have only 2 to 4 weeks in which to review manuscripts, we have also been somewhat taken aback by lengthy response times by journals to our submissions. Elsewhere it has been found that publication is not as delayed as expected [12]. Wallach *et al*. [12] has pointed out that published data on the speed of the publication process has mainly focused on submissions to individual journals and thus do not give an adequate reflection of author experiences regarding submissions and resubmissions. It is against this background that we decided to embark on this study in our setting.

**Aim:** the aim of this study was to describe journal response types and times for manuscripts finalised for journal submission by the School of Medicine (SoM) medical editor, University of the Free State (UFS), from 2014 to 2017.

## Methods

**Study population and sampling:** this descriptive cohort study with an analytical component included all manuscripts finalised for journal submission by the SoM medical editor from 2014 to 2017. These manuscripts were written by health professions professionals and students in the SoM, UFS.

**Data collection:** a data form was compiled by the authors to capture the following information from the medical editor´s Excel spreadsheets containing all the stages and activities of the manuscript process: manuscript and journal characteristics, journal response times and types, need for author intervention to speed up journal response, technical problems experienced with submission platform, number of reviewers involved, number of rounds of feedback from journal until final decision

**Pilot study:** the first submission of each of the four years was included in a pilot study to test the data form and assess the available information. The data form was amended to add a few new items: tracking numbers on medical editor database, nature of contact with journal before submission, whether the manuscript was actually sent for review by the journal and whether the journal editor him/herself gave any feedback. In addition, some options were added or rephrased. The pilot study cases were included in the main study.

**Data analysis:** the data were entered into an Excel spreadsheet. The results are presented by frequencies and percentages (categorical variables), and medians and ranges (numerical variables).

**Ethical consideration:** the protocol was approved by the Health Sciences Research Ethics Committee, UFS (HSREC 143/2017). Permission to conduct the study was obtained from the Head of the SoM, Dean of the Faculty of Health Sciences, Dean of Students Affairs, and Vice-Rector of Research. No details regarding the authors of manuscripts were noted or reported on.

## Results

Ninety-five (n=95) manuscripts were submitted. Due to only 48.4% of manuscripts being accepted by the first journal submitted to, the total number of submissions was 163. Submissions were made to 72 different peer-reviewed, accredited journals: 32 South Africa (SA)-based journals - 104 submissions; 46 journals based elsewhere - 59 submissions. Most of the submissions were full-length articles (87.1%), while the remaining submissions included review articles (5.5%), scientific letters (3.7%), case reports (1.8%), technical notes (1.2%) and an abstract (0.6%). Almost all of the submissions (95.1%) were done through online platforms. There were 13 different online platforms. For six submissions at six journals, the platform changed during the review process. Technical issues regarding platforms (such as a submission that went missing during the transition, fields that were not fully functional, incorrect or incomplete information placed by journal or platforms not functioning correctly) were experienced with 16 (10.3%) platform submissions to 13 journals. Less than 5% of submissions were made to a pre-specified email address. For one journal where one submission was sent electronically, emails, including reviewer feedback, from the journal did not reach the authors.

Table 1 outlines the characteristics of first submissions. First submissions were mainly of undergraduate (36.8%) or postgraduate student projects (35.8%). The vast majority (33/35, 94.3%) of undergraduate manuscripts were submitted to SA-based journals, and the undergraduate manuscripts had the highest percentage (21/35, 60.0%) of first submissions accepted. Less than half of postgraduate student projects and staff projects were accepted by the first journal submitted to. Table 2 summarises the journal response types and times of all submissions. The largest percentage (48.1%) of submissions had 'rejected' as first response from the journal. Median time from submission to first response from the journal was short for rejected or non-compliant submissions, and less than 3 months for the other main response categories. Extreme values of more than a year did occur. Submissions sent for review had a median of 2 reviewers (range 1-3 reviewers). The median time given by the journal for revisions to be made was 1 month (ranging from 1 day to no time specified). In only 15.5% of submissions to be revised did the authors ask for an extension to this timeline. After a revised document had been submitted, journals responded after a median of 13.5 days (range 0-180 days).

**Table 1:** characteristics of first submission (n=95)

Type of project	n (%)	Submitted to SA-based journal	Accepted by first journal submitted to
		n (%)	n (%)
Undergraduate student project	35 (36.8)	33 (94.3)	21 (60.0)
Postgraduate student project	34 (35.8)	21 (61.8)	13 (38.2)
Staff member project	24 (25.3)	13 (54.2)	11 (45.8)
Intern project	2 (2.1)	1 (50.0)	1 (50.0)

SA: South Africa

**Table 2:** response from journal: types and times (n=161^*^)

Response type	n (%)	Median time in days since submission (range)
**First response from journal**		
Non-compliance with requirements	8 (5.0)	2 (0-23)
Rejected	77 (47.8)	15 (0-381)
Resubmission	7 (4.3)	48 (27-346)
Revisions required	59 (36.6)	81 (18-546)
Accepted with corrections	1 (0.6)	174 (0)
Accepted as is	9 (5.6)	88 (0-182)
**Final decision from journal**	**n**	**Median time in days since submission (range)**
Rejected	82	15.5 (0-381)
Accepted	72	119.5 (0-674)

* Two submissions unknown data

Regarding final decisions by journals, rejected submissions had a median response time of just over 2 weeks, and accepted submissions approximately 4 months. Extreme values of more than a year occurred. Only 3.4% of submissions initially requiring revisions were subsequently rejected. Accepted submissions had a median of one round of revision (range 0-4 rounds), and were accepted after a median time of 4 months. The nine submissions not reflected as having a final response consist of eight submissions for which the authors declined to do the proposed resubmission or revisions, and one submission that was erroneously archived by the journal. In 12 submissions (7.4%), there was evidence that author enquiries were made to the journal regarding progress before first journal response. This was done after a median of 147.5 days (range 84-266 days). For five submissions (3.1%), author enquiries were made regarding progress during later review rounds. For three submissions, reviewer feedback or final decision were clarified with the journal. Of the 82 finally rejected submission, 32.5% were sent out for review. The main reasons for rejection was out of scope of journal/not suitable for journal readership (31.2%), methodological issues (30.0%) and no new information (13.8%). For 11.3% of rejected submissions, no reason was given.

## Discussion

As found in our study, it can be anticipated that a manuscript may need to be submitted to numerous journals before acceptance [13]. A call has therefore been made for a universal manuscript format for journal submission [13]. Currently, these different submissions may require time-consuming changes in manuscript structure, layout, technical presentation and referencing style. Undergraduate student projects had the highest percentage of acceptance on the first submission, which could be explained by the level of impact of the journals submitted to. Our findings regarding journal response times compare favourably to those of a survey done among corresponding authors of articles published in December 2016 in Medline indexed journals [12]. That study found the median time from submission to final acceptance 5 months. That study, however, relied on recall of approximate dates and had a response rate of 21%. A review of 37 manuscripts published in the Australasian Medical Journal [14] found that papers were returned to authors for revision on average 1.7 times compared to a median of 1 in our study. Cornelius [14] found a median review time of 74 days, but it is unclear whether this is the journal response time as defined in our study. The extreme isolated first response times of more than a year are ethically unacceptable since during that time the authors cannot submit the material elsewhere, and by the time the journal does respond with a rejection the findings and literature may be outdated.

A study of internal editor review (before peer review) of manuscripts submitted to Academic Medicine [15] found the following nine main themes for internal editor rejection (in order of frequency): ineffective study question and/or design, suboptimal data collection process, weak discussion and/or conclusions, unimportant or irrelevant topic to the journal´s mission, weak data analysis and/or presentation of results, text difficult to follow, to understand, inadequate or incomplete introduction, other publishing considerations, and issues with scientific conduct. The main advice for prospective authors was: 1) finding the right fit between the manuscript and the journal, 2) crafting a clear research question and design, and 3) acting responsibly as a researcher. The Australasian study [14] reported that in a third (35.8%) of papers, instructions to authors were not adhered to, while nearly a third (29.8%) of papers contained major grammatical errors. Garg, Das, and Jain [16] found the main reason for rejection of medical research articles submitted to the Journal of Clinical and Diagnostic Research to be what they call commonality - topics well researched previously, no new perspective or lost relevance (36%), followed by methodological issues (20%), and non-compliance of author (17%). Only 1.5% was rejected as out of scope. The very low percentage of journal response as non-compliance in our study can be ascribed to the diligent work of the medical editor. Our submissions, however, clearly need attention regarding finding the right fit between the manuscript and the journal.

Mun [17] has cautioned that long author delays in submitting revised versions may lead to later rejection. In the Australasian study [14], authors took a median of 22 days per review round to revise the manuscript. In only 15.5% of our submissions did authors require an extension to the journal´s set time period for revision. Garg *et al*. [16] have stressed the importance of author communication with journals. In the cases in our study where there was interaction with journals regarding enquiries regarding submission progress or extension to revision time, journals were responsive and accommodating. Matthews [18] has indicated that journal editors would prefer more frequent interaction with authors. She pointed out that communication between researchers and journals is normally according to certain rigid constraints, which could lead to frustration for both sides. She found that researchers were however uncertain whether discussion with journal editors is appropriate [19]. Most online submission platforms have a field indicating the stage that the submission is in, and we encourage researchers to view this regularly. If no progress is shown within reasonable time (for example, status remains 'awaiting editor assignment' for longer than 6 weeks) it is worth following up with the journal.

**Study limitations:** numerous factors other than those considered in this study can play a role in journal response times. Wallach *et al*. [12] found response time differences in terms of the type of study such as clinical trials, meta-analyses/systematic reviews and observational studies, and postulated that higher-impact factor journals have more rounds of review and therefore longer time to final approval. Some journals (for example those using the platform Editorial Manager) require that co-authors declare their affiliation online after submission before the manuscript is sent for review. Journals have different approaches to the editorial screening of manuscripts before sending them for review. The number of reviewers involved, reviewer response times and frequency of journal publication are further factors that can influence journal response times. These factors are too numerous to investigate in the sample we have, or the necessary information is not readily available. For only one journal was there a sufficient number of submissions to enable a journal specific analysis [20]. The formulation of journal responses was occasionally such that it was difficult to distinguish between a rejection and a request for resubmission.

## Conclusion

Within this setting, the majority of first submissions were unsuccessful, but rejection usually occurred rapidly and only rarely after revisions were requested. Accepted submissions were accepted at a median time of 4 months, after one round of revision. Understanding and awareness of possible expected outcomes and response times could ease some of the tension surrounding the publication process and prevent authors from being vulnerable to predatory journals, who make tantalising claims of rapid publication times.

**Recommendations:** these findings will be of value to the authors in their day-to-day interaction with researchers, to advise them regarding realistic/expected processes, responses and timelines. As described by Balch *et al*. [21]: 'success in scientific writing is dependent on effort, repetition and commitment' and the review process is an educational process. Inexperienced researchers should realise that feedback and criticism are part of the process to improve the quality of the manuscript. Authors should keep detailed records of their submissions to have a realistic picture of the time aspect and when intervening with a journal becomes necessary. A lapse in communication may in fact be due to technical issues regarding the platform of which the journal may be unaware. Experienced authors or subject matter experts should consider being reviewers as a small pool of reviewers may negatively affect turnaround times. Journals can provide a flow diagram of their internal review processes and regular summaries of their response times so that authors can be aware of anticipated timelines at a specific journal and plan accordingly. Journals should be explicit, clear and consistent regarding their scope.

### What is known about this topic

- Publishing is a key aspect of health professionals' lives and seemingly often fraught with obstacles;
- Published data on the speed of the publication process has mainly focused on submissions to individual journals.

### What this study adds

- Quantified data regarding submissions and resubmissions over a broad spectrum of medical and health profession journals;
- Such quantified data regarding expected outcomes and response times and the practical guidelines provided can help researchers to plan appropriately for the publication process and prevent authors from being vulnerable to predatory journals, who make tantalising claims of rapid publication times.
